# 1-Octen-3-ol – the attractant that repels

**DOI:** 10.12688/f1000research.6646.1

**Published:** 2015-06-18

**Authors:** Pingxi Xu, Fen Zhu, Garrison K. Buss, Walter S. Leal

**Affiliations:** 1 Department of Molecular and Cellular Biology, University of California-Davis, Davis, CA, 95616, USA

**Keywords:** Culex quinquefasciatus, odorant receptors, chiral discrimination, antennae, maxillary palps, CquiOR118b, CquiOR114b, repellency assay

## Abstract

Since the discovery in the early 1980s that 1-octen-3-ol, isolated from oxen breath, attracts tsetse fly, there has been growing interest in exploring the use of this semiochemical as a possible generic lure for trapping host-seeking mosquitoes. Intriguingly, traps baited with 1-octen-3-ol captured significantly more females of the malaria mosquito, *Anopheles gambiae*, and the yellow fever mosquito, *Aedes aegypti, *than control traps, but failed to attract the southern house mosquito, *Culex quinquefasciatus*. Additionally, it has been demonstrated that this attractant is detected with enantioselective odorant receptors (ORs) expressed only in maxillary palps. On the basis of indoor behavioral assays it has even been suggested that 1-octen-3-ol might be a repellent to the southern house mosquito. Our approach was two-prong, i.e., to isolate 1-octen-3-ol-sensitive ORs expressed in maxillary palps and antennae of southern house female mosquito, and test the hypothesis that this semiochemical is a repellent. An OR with high transcript levels in maxillary palps, CquiOR118b, showed remarkable selectivity towards ( *R*)-1-octen-3-ol, whereas an OR expressed in antennae, CquiOR114b, showed higher preference for ( *S*)-1-octen-3-ol than its antipode. Repellency by a surface landing and feeding assay showed that not only racemic, but enantiopure ( *R*)- and ( *S*)-1-octen-3-ol are repellents at 1% dose thus suggesting the occurrence of other ( *S*)-1-octen-3-ol-sensitive OR(s). Female mosquitoes with ablated maxillary palps were repelled by 1-octen-3-ol, which implies that in addition to OR(s) in the maxillary palps, antennal OR(s) are essential for repellency activity.

## Introduction

**Figure 1.**
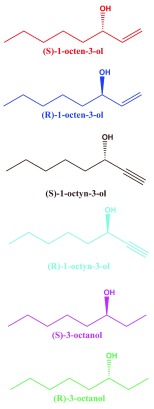
Chemical structures of tested compounds. ( *S*) and ( *R*)-1-octen-3-ol, ( *S*) and ( *R*)-1-octyn-3-ol, ( *S*) and ( *R*)-3-octanol.

1-Octen-3-ol ( Figure 1) is a natural product derived from linoleic acid, which was first isolated from the matsutake pine mushroom ^1^ and thereafter from plants and other fungi. It is approved by US Food and Drug Administration (ASP 1154, Regnum 172.515) as a food additive and also considered a wine fault – an unpleasant characteristic of wine. Since it was discovered as an emanation from oxen breath that attracts tsetse fly ^2^, there has been growing interest in using 1-octen-3-ol as an insect attractant. Indeed, it was demonstrated earlier on that 1-octen-3-ol synergizes with CO _2_ and thus increase mosquito trapping efficacy ^3^. Intriguingly, field experiments demonstrated that the effect of 1-octen-3-ol on mosquito captures is species specific ^4^. Of notice, 1-octen-3-ol seem to have little or no effect on trapping of the southern house mosquito, *Culex quinquefasciatus*, although being undoubtedly an attractant (kairomone) for *Anopheles* and *Aedes* mosquitoes ^4^.

Club-shaped olfactory basiconic sensilla (peg sensilla) in the maxillary palps of the southern house mosquito harbor three types of olfactory receptor neurons (ORNs) identifiable by their spike amplitudes ^5^. The second largest neurons, ORN-B, responded to 1-octen-3-ol with very high sensitivity. Cell B also showed a remarkable selectivity between the two enantiomers of 1-octen-3-ol, with the ( *R*)-(-)-isomer eliciting robust responses at 10 ng dose (256.6 ± 12 spikes/s), whereas the ( *S*)-(+)-antipode eliciting only 115.5 ± 23 spikes/s even when challenged with 100x higher does, i.e., 1 µg ^5^. It was also demonstrated that neuron-B in the maxillary palps of the malaria mosquito, *Anopheles gambiae*, responds to 1-octen-3-ol ^6^, and chiral discrimination was also observed with electrophysiological recordings from the neuron B in the maxillary palps of the yellow fever mosquito, *Aedes aegypti* ^7, 8^. Additionally, odorant receptors housed in the maxillary palps of the malaria mosquito ^6^ and yellow fever mosquito ^9^, AgamOR8 and AaegOR8, respectively, showed significant preference for the ( *R*)-enantiomer when co-expressed in *Xenopus* oocytes along with the obligatory co-receptor Orco.

In-door behavioral studies demonstrated that at two doses ( *R*)-(-)1-octen-3-ol caused an increase in activation for *Cx. quinquefasciatus*, and at seven of the doses tested ( *R:S*)-1-octen-3-ol mixture (84:16) caused significantly more mosquitoes to sustain their flight and reach the capture chambers in a two-choice, Y-tube olfactometer thus suggesting that the isomeric mixture has an excitatory effect ^7^. Additionally, they observed that at the highest concentration, mosquitoes that reached the capture chambers moved towards the control chamber rather than the arm containing ( *R*)-(-)-1-octen-3-ol per se or in mixtures, i.e., a reduced attraction response mediated by the ( *R*)-enantiomer ^7^. Since the ability of the olfactory system to detect the two enantiomers at this close ratio (approximately 5:1) was not observed in our electrophysiological recordings from peg sensilla ^5^, we aimed at testing chiral discrimination at the receptor level. Using cDNA template from the maxillary palps, we cloned the C *ulex* ortholog of AgamOR8 and AaegOR8, co-expressed it along with CquiOrco in *Xenopus* oocytes, and observed an ability to discriminate enantiomers that reflects our previous findings with single sensillum recordings. Additionally, we cloned a paralogous odorant receptor (OR) from antennae, which responded to both enantiomers of 1-octen-3-ol. These findings provide evidence that peripheral reception of 1-octen-3-ol is enantioselective at the maxillary palps, but random (racemic) at the antennae.

## Materials and methods

### cDNA preparation

*Culex quinquefasciatus* mosquitoes used in this study were from a laboratory colony ^10^, maintained for the last 5 years at 27 ± 1°C under a photoperiod of 12:12 h (light:dark). Our Davis colony was derived from mosquitoes collected in Merced, California, in the 1950s and maintained by Dr. Anthon Cornel in the Kearney Agricultural Center, University of California. Twenty pairs of antennae and maxillary palps of 9-day old gravid female adults were dissected on ice under a light microscope. Total RNA was extracted using RNeasy Micro Kit (QIAgen, Valencia, CA). Before synthesizing first-strand cDNA, RNA concentrations from antennae and maxillary palps extracts were adjusted (normalized). First-strand cDNA was synthesized with oligo (dT) primer (BioRad, Hercules, CA) and GoScript Reverse Transcriptase (Promega, Madison, WI) following the manufacturer’s protocol.

### Gene cloning

Full-length sequences of CquiOR114b and CquiOR118b were amplified from female antennae and maxillary palps cDNAs, respectively. The In-Fusion cloning strategy was taken by using In-Fusion ^®^ HD Cloning Kit (Clontech ^®^ Laboratories, Mountain View, CA). Briefly, the PCR primers were designed with 16 overlapped nucleotides at 5’-end homologous to the linearized ends of the destination vector (pGEMHE), which was double digested by *XbaI* and *XmaI*. The primers for CquiOR114b were: forward, **AGATCAATTCCCCGGG** **acc**ATGGCTACGAAGAAGGTTGCATTC; and two reverse primers, reverse-1: **TCAAGCTTGCTCTAGA**TTACGATCCTTCATAAACCGCCTT and reverse–2: **TCAAGCTTGCTCTAGA**TTACAACTCAAAGGAAACTCTGCTAACTCC. Low case “acc” stands for Kozak sequence.

For CquiOR118b two forward and one reverse primers were used; forward-1: **AGATCAATTCCCCGGG**ATGAACGACCTGGTGCGGTTCGAG and forward-2: **AGATCAATTCCCCGGG**ATGCATGTGGGCAACTCCAAGATTTCG; reverse, **TCAAGCTTGCTCTAGA**TTATTTCTCGCTGGGATCATAAATAGTTTTCAGCAG. Underline denotes homologous sequence for In-Fusion reaction. PfuUltra II Fusion HS DNA Polymerase (Agilent Technologies, Santa Clara, CA) was used for PCR. PCR products were directly cloned into pGEMHE by using In-Fusion ^®^ HD Cloning Kit, following the manufacturer’s protocol. In brief, a mix of PCR product, In-Fusion HD Enzyme Premix, pGEMHE vector was incubated at 50°C for 15 min. One microliter of the reaction was added to Stellar™ competent cells for transformation. Plasmids were purified by plasmid mini prep columns SpinSmart (Denville Scientific, South Plainfield, NJ) and sequenced by Davis Sequencing Inc. (Davis, CA).

### Quantitative RT-PCR (qPCR)

SsoAdvanced™ SYBR ^®^ Green from BioRad was used for qPCR. The reactions were carried out in a BioRad C1000 thermal cycler with CFX96 detection module. The detection primers for CquiOr114b were: forward, TTAGCGGGA GAAAACATGGG; reverse, ACTGACTTTGGTACAC GTGG. For CquiOr118b, they were: forward, GTCGTTGCTTTTCCTGATGG; reverse, CACGGCATT CTCATATTTTACACT. The following primers were used for a reference gene, *CquiOrco*: forward, GCCGGATACGTTTTCTCCTTC; reverse, GCGCATAATTCCCTTCAGATG. The reaction system (total volume, 20 µl) included SsoAdvanced SYBR green mix (2x) 10 µl, cDNA 100 ng, paired primer mix 350 nM, and double distilled H _2_O. The qPCR program was 95°C for 30 s, 95°C for 5 s, 62°C for 30 s, 72°C for 30 s, and 40 cycles. The melt curves were made from 65°C to 95°C with increment of 5°C, 5 s.

## Electrophysiology

### Two-Electrode Voltage Clamp Records

The two-electrode voltage-clamp (TEVC) technique was used to measure odorant-induced currents in *Xenopus* oocytes at a holding potential of −80 to −70 mV. Oocytes on stage V or VI were purchased from Ecocyte Bioscience (Austin, TX). Signals were amplified with an OC-725C amplifier (Warner Instruments), low-pass–filtered at 50 Hz, and digitized at 1 kHz. Data acquisition and analysis were conducted with Digidata 1440A and software pCLAMP10 (Molecular Devices). Traces were collected from same batches and same age of oocytes to make data consistent. Data were analyzed with GraphPad Prism 6 (La Jolla, CA). The following chiral compounds were gifts from Bedoukian Research Inc.: ( *R*)-(-)-1-octen-3-ol (CAS# 3687-48-7), ( *S*)-(+)-1-octen-3-ol (CAS# 24587-53-9); ( *R*)-(-)-1-octyn-3-ol (CAS#32556-70-0), ( *S*)-(+)-1-octyn-3-ol (CAS#322556-71-1); ( *R*)-(-)-3-octanol (CAS#70492-66-9). Racemic 1-octen-3-ol (CAS # 3391-86-4) and ( *S*)-(+)-3-octanol (CAS# 22658-92-0) were acquired from Fluka and Aldrich, respectively (Sigma-Aldrich, Milwaukee, WI).

### Behavioral assays

Repellence was measured by using a previously described surface-landing and feeding assay ^10^. In short, 3–5 days-old female mosquitoes (30–40 mosquitoes per assay) were placed on a two-choice arena designed to attract host-seeking mosquitoes. For physical stimuli, water at 37°C was circulating inside of Dudley tubes, which were painted black in the internal surfaces. Chemical stimuli were provided by stream of CO _2_ at 50 ml/min and dental cotton rolls impregnated with defibrinated sheep blood, which were placed on the top of the Dudley tubes. For each test, filter paper rings freshly treated at the outer perimeter with 200 μl of hexane only or 200 μl of a tested compound in hexane were placed to surround each Dudley tube. Mosquito activity was observed and recorded for 5 min with a camcorder equipped with Super NightShot Plus infrared system (Sony Digital Hanycam, DCR-DVD 810). Control and treatment sides were rotated between trials. For all experiments except the concentration screen (used twice), the rings were prepared fresh for each assay. The number of mosquitoes responding to control (hexane only) and treatment were counted in real time and the information also retrieved from video recordings. When testing repellency by racemic and enantiomers, experiments were carried out by using all three compounds, R, S, and racemic in a single set of assays. Each compound was tested interactively, such that R was followed by S and S was followed by racemic, giving rise to a “block” of 3 trials. Three blocks (n=9 for each compound) were conducted per assay. In about half of the trials (n=9 repetitions per compound) test compounds were placed in one of the two sides of the arena. Data were arcsin-transformed before paired two-tailed Student t test comparisons.

## Results and discussion

Raw data Dataset 1: qPCR data obtained for *CquiOR114b*, *CquiOR118b*, and *CquiOrco* (reference gene) with cDNAs from antennae and maxillary palps.
Dataset 2: Concentration-response data generated with CquiOR118b and enantiomers of 1-octen-3-ol, 1-octyn-3-ol, and 3-octanol.
Dataset 3: Concentration-response data generated with CquiOR114b and enantiomers of 1-octen-3-ol, 1-octyn-3-ol, and 3-octanol.
Dataset 4: Data for repellency activity elicited by 1-octen-3-ol and its enantiomers on female *Culex quinquefasciatus* in a surface-landing and feeding assays. Click here for additional data file. Copyright: © 2015 Xu P et al. 2015 Data associated with the article are available under the terms of the Creative Commons Zero "No rights reserved" data waiver (CC0 1.0 Public domain dedication).

### Cloning and tissue expression

We aimed at cloning CquiOR118, the *Cx. quinquefasciatus* ortholog of AgamOR8 and AaegOR8. Despite several attempts, we were unable to clone the full length cDNA (VectorBase, CPIJ013954). On the basis of our RNA-Seq findings suggesting a shorter N-terminal amino acid sequence ^11^, we designed a new forward primer considering the starting codon as the next ATG. Indeed, this led to the full length sequence, which was cloned and confirmed by DNA sequencing. We named the shorter version of this gene *CquiOR118b*, which encodes a protein with 391 amino acid residues and is predicted to have seven transmembrane topology (OCTOPUS, http://octopus.cbr.su.se/). While this manuscript was in preparation it has been reported ^2^ that a longer version of CquiOR118, as predicted in VectorBase, was cloned from another strain of *Cx. quinquefasciatus.* Thus, both CquiOR118b from the Merced strain (see below) and CquiOR118 from the Thai strain are functional ^2^. Out of 5 clones we sequenced, we obtained two isoforms of CquiOR118b, which differed only in the residue 163 predicted to be in the external cellular loop-2: Ile vs. Val.

Our previous differential expression analysis ^11^ suggested that transcript levels of two genes from the same clade, *CquiOR114* and *CquiOR117*, are significantly higher in antennae than control tissues (legs). Because of the predicted longer C-terminus amino acid sequences encoded by these genes ^11^, we designed primers that would allow us to clone the short and longer versions of these genes. No PCR product was generated with primers for the short sequences, but we cloned and sequenced cDNAs (CquiOR114b) encoding proteins with 405 amino acid residues (longer C-terminus) and predicted seven transmembrane topology. Out of 5 cloned sequenced, we found two isoforms, which differed in 3 amino acid residues. We named them CquiOR114b-1 (Leu-63, Gly-122, and Asp-129) and CquiOR114b-2 (Trp-63, Glu-122, and Asn-129). These residues are predicted to be part of the first transmembrane segment formed by residues 60 to 80, and the internal cellular loop-2 formed by residues 112–150.

**Figure 2.**
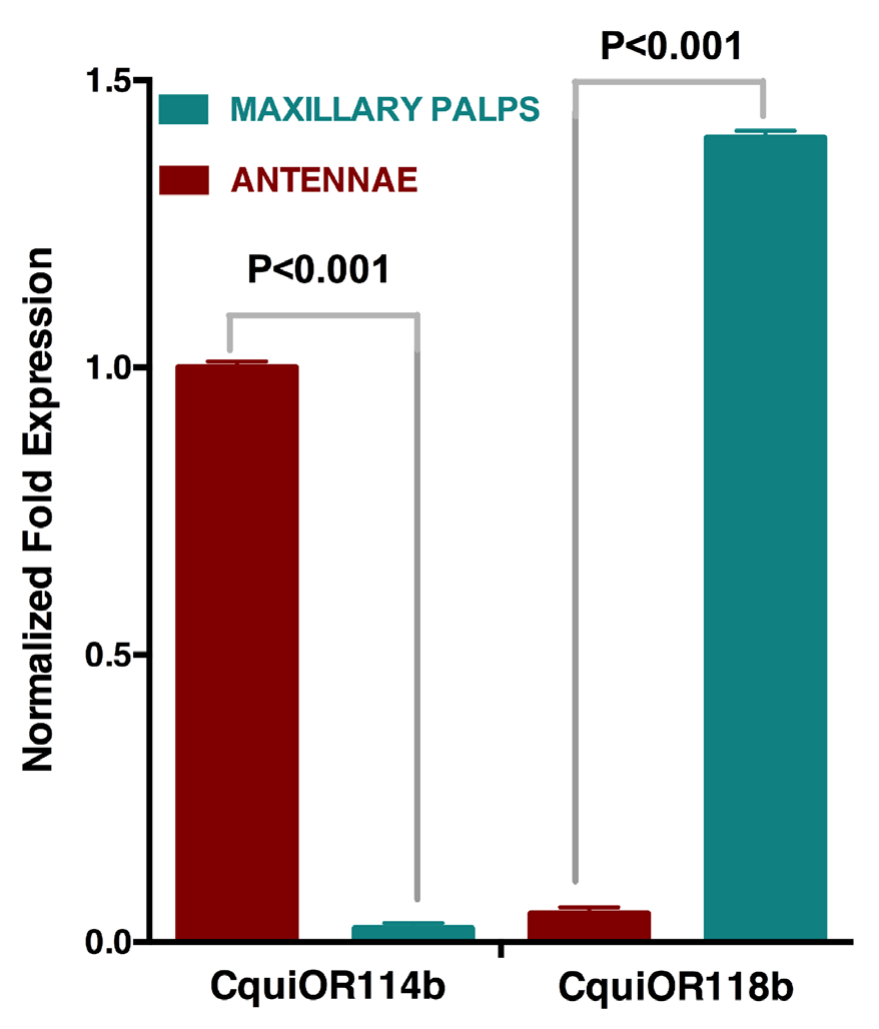
qPCR analysis of newly cloned receptors. Data show high transcription levels of *CquiOR114b* and *Cqui118b* in antennae and maxillary palps, respectively.

Quantitative PCR analysis showed that indeed *CquiOR114b* was expressed in antennae but not in the maxillary palps, whereas *CquiOR118b* was expressed in the maxillary palps but not in antennae ( Figure 2). Next, we used the *Xenopus* oocyte recording system to compare the responses of the newly cloned ORs and their isoforms.

### Chiral discrimination by CquiOR118b

**Figure 3.**
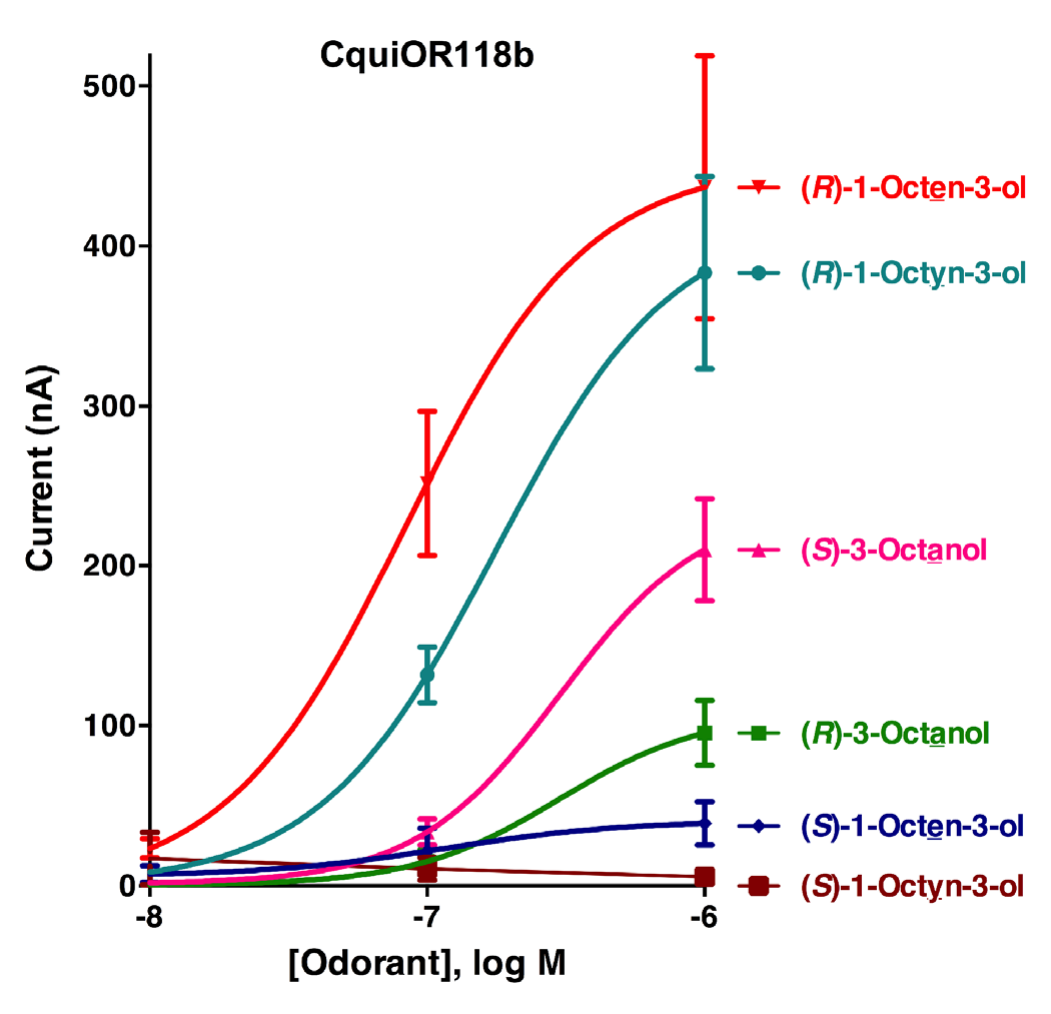
Concentration-response relationships for CquiOR118b. CquiOR118b-CquiOrco-expressing oocytes were challenged with enantiomers of 1-octen-3-ol, 1-octyn-3-ol, and 3-octanol at 0.01, 0.1, and 1 µM doses. (N = 3)

Initially, the responses of oocytes co-expressing one of the isoform of CquiOR118 and the obligatory co-receptor CquiOrco were compared. Since there were no significant difference in the responses elicited by Ile-163-CquiOR118b and Val-163-CquiOR118b, we used only colony 1 (GenBank, KT022418) in subsequent analysis. Next, we challenged CquiOR118b·CquiOrco-expressing oocytes with enantiomers of 1-octen-3-ol and C8 analogs, namely, 1-octyn-3-ol and 3-octanol. While robust responses were elicited by ( *R*)-1-octen-3-ol in a dose-dependent manner, currents generated by its antipode, ( *S*)-1-octen-3-ol, were relatively very small ( Figure 3). The remarkable ability of CquiOR118b expressed in a heterologous system to discriminate enantiomers of 1-octen-3-ol is in line with the observations with the intact olfactory system ^5^. Likewise, CquiOR118b showed dramatic enantioselectivity towards ( *R*)-as compared to ( *S*)-1-octyn-3-ol. The receptor showed reduced selectivity towards the saturated analog, 3-octanol. Although at higher doses it preferred ( *S*)-3-octanol, the responses to ( *R*)-3-octanol were relatively high. It is worth mentioning that the hydroxyl group in the ( *S*)-enantiomer of the saturated analog has the orientation as in the ( *R*)-isomers of the unsaturated counterparts ( Figure 1), their nomenclature differing ( *S* vs. *R*) because of the IUPAC rules, not the orientation of the polar moiety expected to fit in the binding cavity of the receptor. Our findings suggest that with CquiOR118b per se the mosquito olfactory system is unlikely to be able to detect the behaviorally relevant ratio of the isomers of 1-octen-3-ol, i.e., R/S, 84:16 ^7^. It is, therefore, likely that 1-octen-3-ol is also detected by other receptor(s).

### Random reception by CquiOR114b

**Figure 4.**
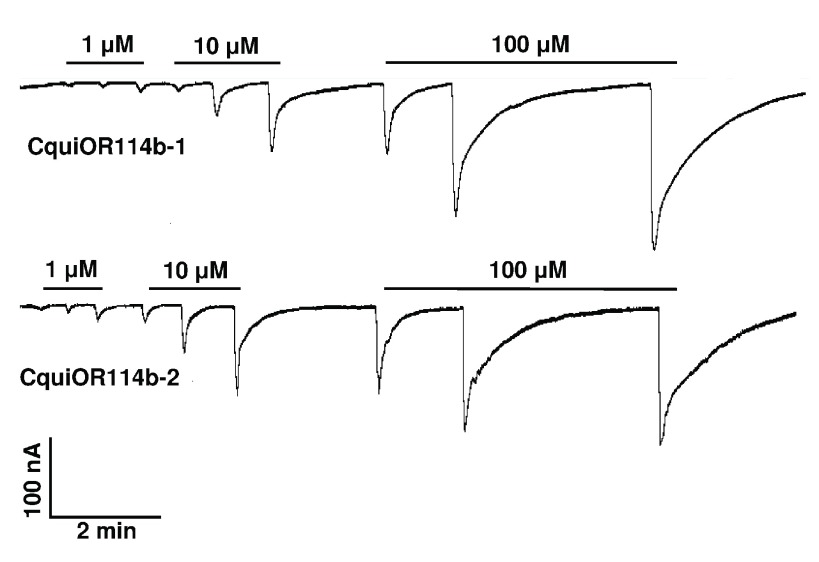
Traces obtained with oocytes co-expressing CquiOR114b-1 and CquiOrco (top trace) and CquiOR114b-2 and CquiOrco (lower trace). Compounds were delivered in the following order: 1-octyn-3-ol, 1-octen-3-ol, and 3-octanol from 1 to 10 µM (left to right).

We first compared the two isoforms of CquiOR114b by challenging with 1-octen-3-ol, 1-octyn-3-ol, and 3-octanol oocytes expressing each isoform, CquiOR114b-1 (GenBank, KT022419) or CquiOR114b-2 (GenBank, KT022420) along with CquiOrco ( Figure 4). Traces comparing these ligands at three different doses were almost indistinguishable, with the responses recorded from CquiOR114b-1·CquiOrco-expressing oocytes being slightly higher than those from CquiOR114b-2. We, therefore, used CquiOR114b-1 to obtain dose-dependent curves.

**Figure 5.**
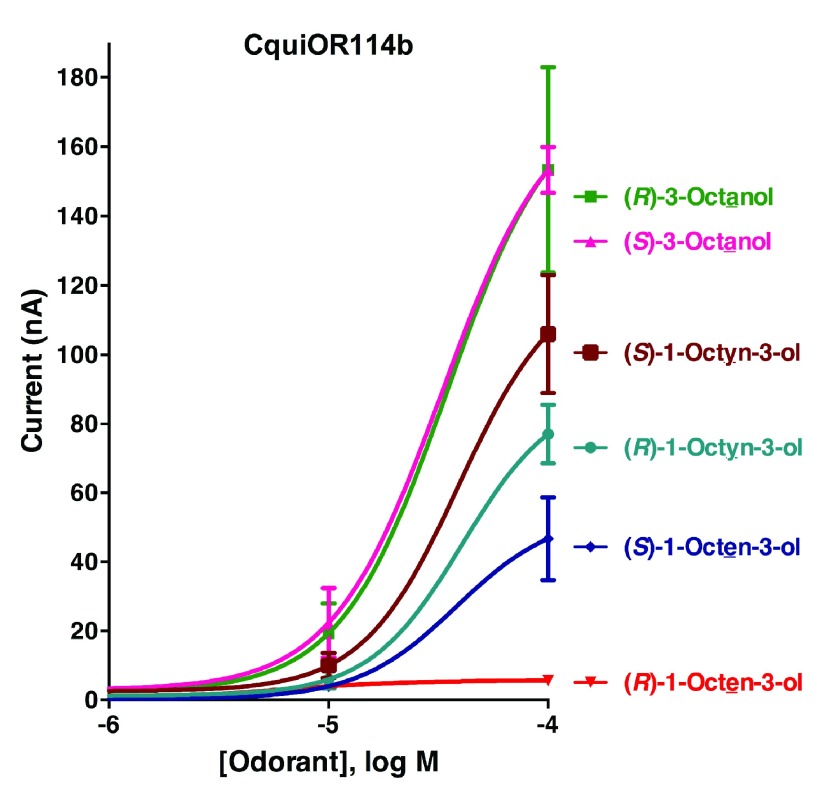
Concentration-response relationships for CquiOR114b. CquiOR118-CquiOrco-expressing oocytes were challenged with enantiomers of 1-octen-3-ol, 1-octyn-3-ol, and 3-octanol at 1, 10, and 100 µM doses. (N = 3)

CquiOR114b·CquiOrco-expressing oocytes gave robust responses to 3-octanol, with responses to the ( *R*)- and ( *S*)-stereoisomers being almost indistinguishable ( Figure 5). Likewise Cqui114b responded to the unsaturated compounds, with a slightly preference for ( *S*)-isoforms. Of notice, currents elicited by ( *R*)-1-octen-3-ol were significantly lower than those obtained with its antipode, ( *S*)-1-octen-3-ol, particularly at 0.1 mM ( Figure 5). It is, therefore, likely that this antennal receptor contributes to the overall reception of ( *S*)-enantiomers of unsaturated C8 alcohols.

### Behavioral responses

**Figure 6.**
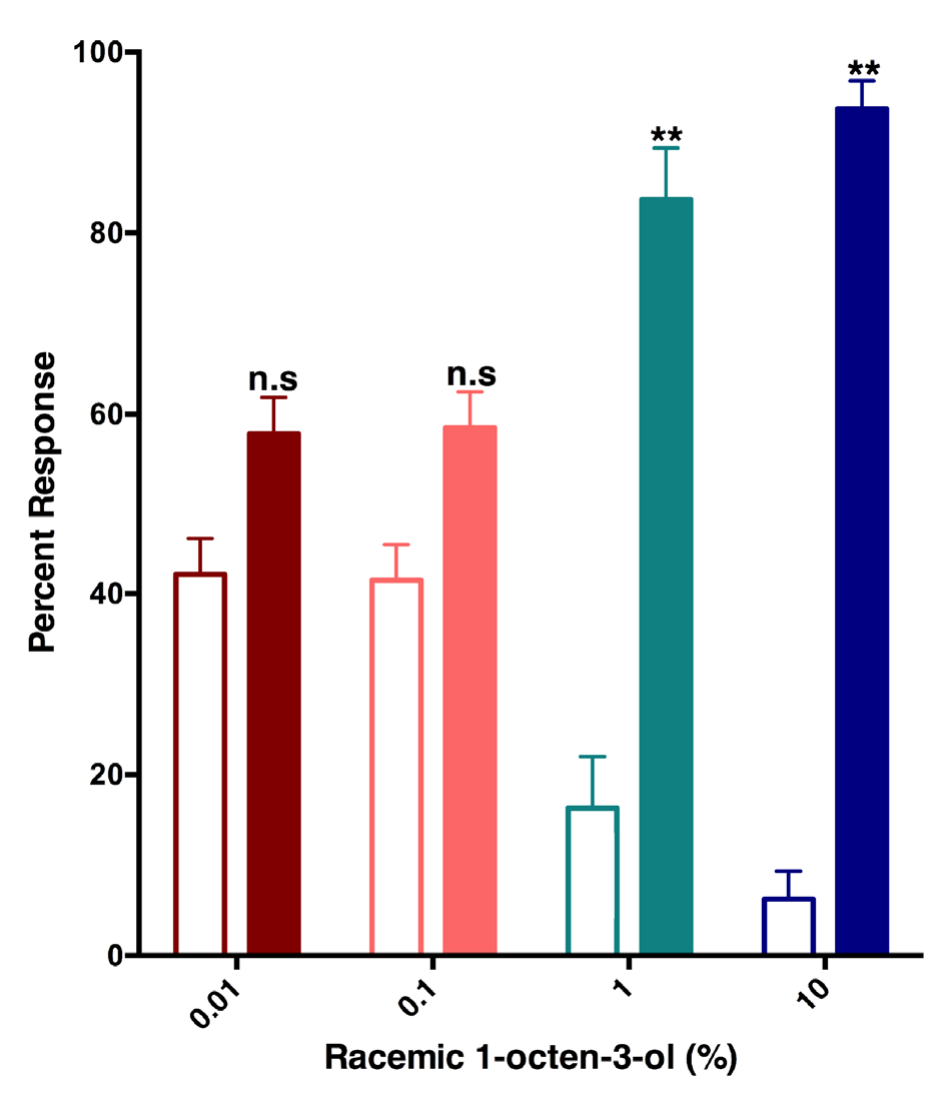
Female *Cx. quinquefasciatus* are repelled by 1-octen-3-ol. In the surface-landing and feeding assay, females of the southern house mosquito were significantly repelled by racemic 1-octen-3-ol at 1 and 10% doses, but not at lower doses. Filled bars represent control.

**Figure 7.**
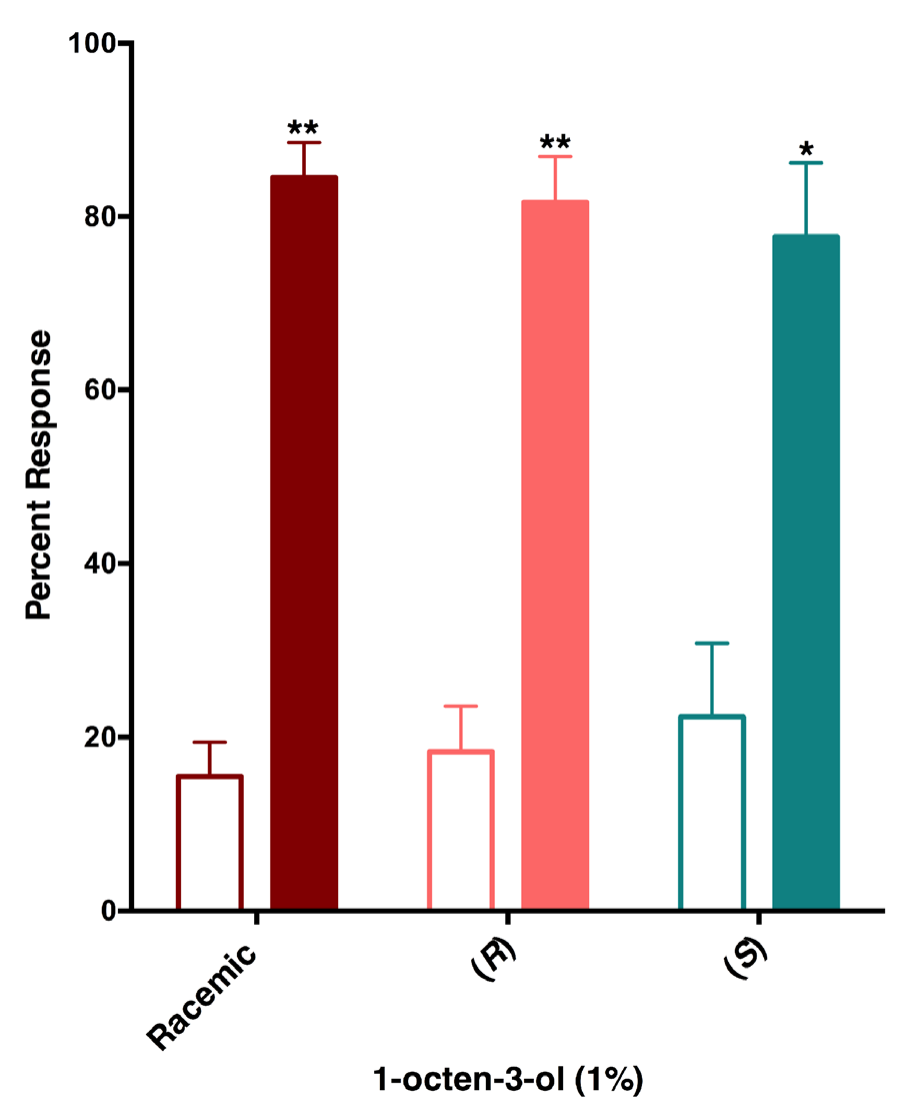
The southern house mosquito is repelled by both enantiomers of 1-octen-3-ol. Female mosquitoes were repelled not only by racemic but also enantiopure isomers, ( *R*)- and ( *S*)-1-ocen-3-ol at 1% dose. Filled bars represent control.

Previously, Cook and collaborators observed an intriguing reduced relative attraction response elicited by ( *R*)-1-octen-3-ol in Y-tube olfactometer, but they were unable to conclude if the effect was true repellency as the design of their arena did not allow repellency measurement. With a recently designed surface landing and feeding assay ^10^, we tested the hypothesis that 1-octen-3-ol is a repellent. Although at very low concentrations of racemic 1-octen-3-ol (0.01 and 0.1%) ( Figure 6) mosquitoes were attracted to both sides of the arena, at higher doses (1 and 10%) they were repelled by 1-octen-3-ol. Next, we compared repellency elicited by enantiomers and racemic 1-octen-3-ol. Surprisingly, both ( *R*)- and ( *S*)-1-octen-3-ol were repellent at the 1% dose ( Figure 7). We then surmised on the basis of dose dependence curves obtained with CquiOR118b ( Figure 3) that other odorant receptor(s) must mediate repellency elicited by ( *S*)-1-octenol-3-ol, possible candidates being CquiOR114b, which we identified from antennae and the recently reported CquiOR113 from maxillary palps ^12^.

**Figure 8.**
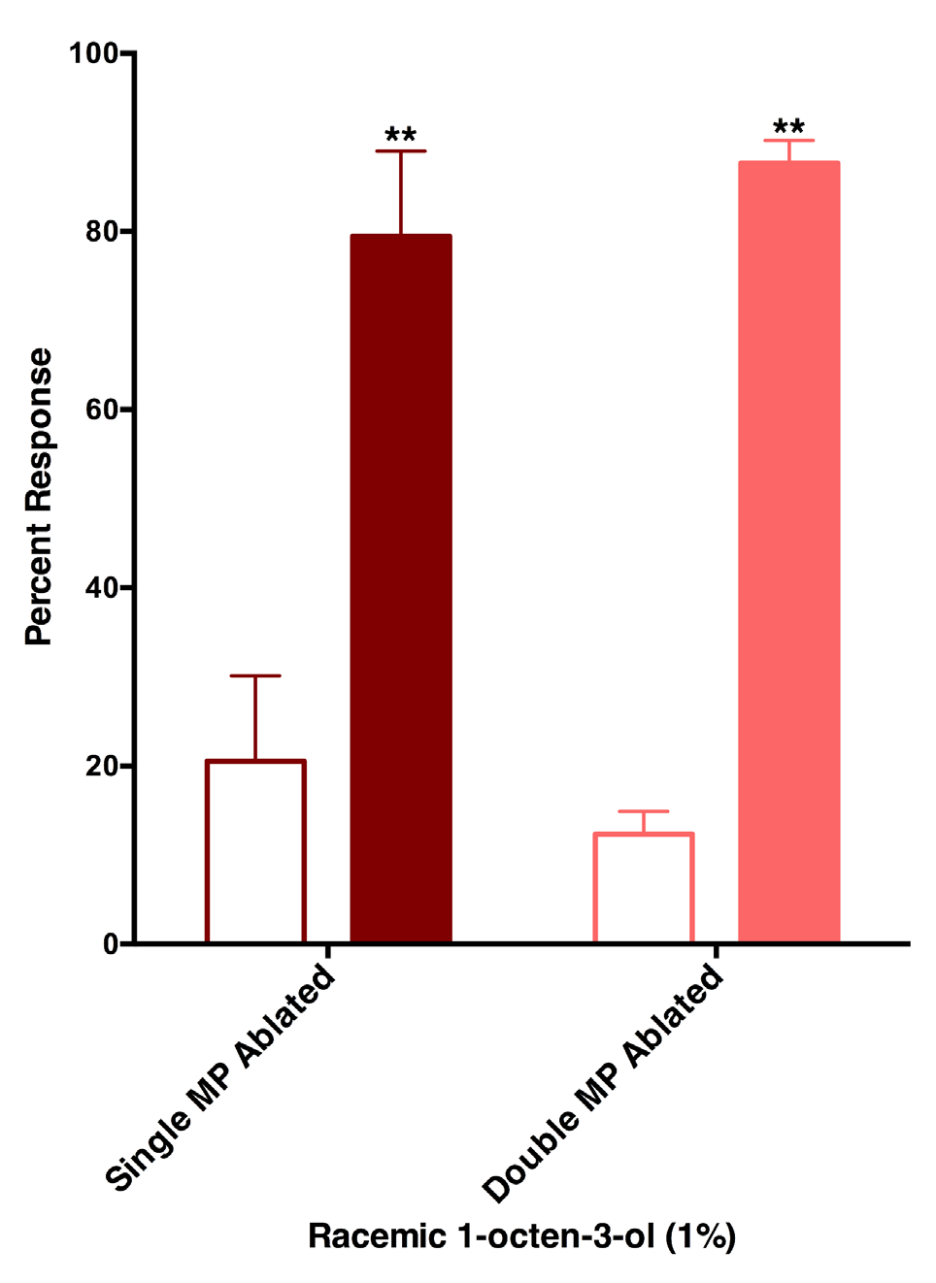
Mosquito with ablated maxillary palps are repelled by 1-octen-3-ol. The effect of surgery on response of female *Culex* mosquitoes to 1-octen-3-ol was minimal given that mosquitoes with one or two maxillary palps ablated were repelled by 1-octen-3-ol. Filled bars represent control.

We then attempted to combine surgery with behavioral measurement to determine if the maxillary palps are the only olfactory tissues involved in reception of this repellent. Mosquitos with ablated antennae show little or no flight activity. It might be that impairing a significant component of the olfactory system, and possibly hygroscopic and thermal detectors, may render mosquitoes completely inactive. By contrast, ablating one or two of the maxillary palps had little effect on mosquito activity. Interestingly, mosquitoes with single or double ablated maxillary palps were still repelled by 1-octen-3-ol ( Figure 8). We, therefore, concluded that the maxillary palps are not sufficient for repellency by 1-octen-3-ol. Other appendages, most likely antennae, are involved in the reception of this repellent.

## Conclusion

We have isolated and cloned two odorant receptors from the southern house mosquito sensitive to 1-octen-3-ol and related compounds. CquiOR118b, which is expressed in the maxillary palps, showed remarkable selective and sensitivity towards ( *R*)-1-octen-3-ol and the related alkyne, ( *R*)-1-octnyl-3-ol. To a much lower extent, CquiOR118b-CquiOrco-expressing oocytes discriminated enantiomers of 3-octanol. By contrast, antennal CquiOR114b responded equally to enantiomers of 3-octanol and showed preference for ( *S*)-isomers of 1-octen-3-ol and 1-octyn-3-ol. Repellency assays showed that both isomers of 1-octen-3-ol, a known attractant for *Anopheles* and *Aedes* mosquitoes, were indeed repellents to *Cx. quinquefasciatus.* However, the maxillary palps alone are not enough for detection of this repellent.

## Data availability

The data referenced by this article are under copyright with the following copyright statement: Copyright: © 2015 Xu P et al.

Data associated with the article are available under the terms of the Creative Commons Zero "No rights reserved" data waiver (CC0 1.0 Public domain dedication). 

F1000Research: Dataset 1. Raw data, 10.5256/f1000research.6646.d49878 ^13^
