# Supplementary material for: 1-Octen-3-ol – the attractant that repels
Source: F1000Res. 2015 Jun 18;4:156. [Version 1] doi: 10.12688/f1000research.6646.1 (PMC4623779; doi:10.12688/f1000research.6646.1)
Supplement: Peer review history

# Peer review: Referee response for version 1

**DOI:** 10.5256/f1000research.7140.r9111

**Published:** 2015-07-02

**Authors:** Kenneth F. Haynes

**Affiliations:** 1 Department of Entomology, University of Kentucky, Lexington, KY, USA

This is a first report that connects olfactory receptors to 1-octen-3-ol in a *Culex* mosquito to behavioral response. In addition, both enantiomers were shown to be repellents. Interestingly the response in the *Culex quinquefasciatus *contrasts with that seen in *Anopholes* and *Aedes* mosquitoes. The adaptive explanation for the contrast between species remains to be explained. This paper reports many important steps towards understanding the mechanisms underlying the behavioral response.

I have read this submission. I believe that I have an appropriate level of expertise to confirm that it is of an acceptable scientific standard.

# Peer review: Referee response for version 1

**DOI:** 10.5256/f1000research.7140.r9110

**Published:** 2015-07-02

**Authors:** Wynand van der Goes van Naters

**Affiliations:** 1 School of Biosciences, Cardiff University, Cardiff, UK

Xu *et al*. report experiments on the reception and behavioral response in the southern house mosquito *Culex quinquefasciatus* to enantiomers and analogs of 1-octen-3-ol. Specifically, the authors find that the maxillary palp receptor Or118b is especially sensitive to the R(-) enantiomer while the antennal receptor Or114b mediates stronger responses to the S(+) than to the R(-) enantiomer. The authors show that both enantiomers repel female *Cx. quinquefasciatus*, as does a racemic mixture. Females in which the maxillary palps have been ablated are similarly repelled by racemic 1-octen-3-ol, proving that the maxillary palps are not solely responsible for the response to this chemical. The paper makes an important contribution within the context of the literature on insect reception of 1-octen-3-ol, which is an attractive kairomone for several haematophagous insects. Experiments are compelling and the conclusions follow from the data. I have only a few minor suggestions:

1. Could the authors please provide more detail on the stimulus method in the oocyte recordings? How long was each stimulus pulse? Black lines above traces sometimes indicate the timing of the stimulus pulse, but this is not the case in Figure 4.
2. Legend of Figure 4: please check the concentration range (to 100 micromolar).
3. Legend of Figure 5, below the title of the legend, starts with "CquiOR118..." instead of "CquiOR114..."
4. Please indicate the meaning of the error bars in the figures (standard deviation or SEM or CI?).
5. While the reader can infer that Or118 was identified by BLAST as an ortholog of Or8 from *An. gambiae*, it is not obvious how Or114 was identified. Could the authors perhaps include a paragraph on the bioinformatics that underlies this work?
6. It is interesting that 1-octen-3-ol repels this mosquito species, but is attractive to several other species. Is there a possibility that 1-octen-3-ol could be attractive for *Culex quinquefasciatus* when combined with other chemicals? 

I have read this submission. I believe that I have an appropriate level of expertise to confirm that it is of an acceptable scientific standard.

# Peer review: Referee response for version 1

**DOI:** 10.5256/f1000research.7140.r9109

**Published:** 2015-07-01

**Authors:** Jeffery K. Tomberlin

**Affiliations:** 1 Department of Entomology, Texas A&M University, College Station, TX, USA

I very much enjoyed reading this article. And, I believe it makes a significant positive contribution to our understanding of mosquito responses to volatiles associated with hosts. I believe the title and abstract are appropriate for the publication. 

With regards to the experiment design, were the trials conducted on the same day from the same population of mosquitoes (could there be a generation effect)? Would the authors consider using logistic regression to analyze the data? Such an approach would determine if there is an interaction between dose, response, and trial?

Furthermore, was there much movement between the treatments (i.e., were mosquitoes flying from one treatment to the next)? If so, how did these responses vary across concentrations?  These data would determine if there was increased activity as a response to the treatment.

Another interesting aspect of the study would be to look at the raw data in conjunction with percent response as these data would also lend towards appreciating the compound "exciting" the mosquitoes.

The discussion and conclusions are well developed. One aspect that would be important to consider is the relationship between the volatile and its source (most likely a fungus) as this compound operates similarly to quorum sensing molecules (concentration of the compound dictates "behavior" of the microbe). By building a bridge between the role of the compound with its source, one would be able to tie together the ecology of the mosquito with the source (i.e., microbe) and host health. Such an approach could provide greater explanation as to why certain doses are attractant while others are repellent.

I have read this submission. I believe that I have an appropriate level of expertise to confirm that it is of an acceptable scientific standard.

# Peer review: Referee response for version 1

**DOI:** 10.5256/f1000research.7140.r9112

**Published:** 2015-06-29

**Authors:** Joseph Dickens

**Affiliations:** 1 Invasive Insect Biocontrol and Behaviour Laboratory, United States Department of Agriculture, Beltsville, MD, USA

The authors detail studies aimed at understanding the olfactory detection of 1-octen-3-ol in the southern house mosquito, *Culex quinquefasciatus*. Behavioral studies showed mosquitoes to be repelled by high concentrations of 1-octen-3-ol. An odorant receptor (OR) identified in the maxillary palps responded selectively to ( *R*)-1-octen-3-ol in a manner similar to ORs in other mosquitoes. However, when the palps were ablated, the repellent effects of octenol at high concentrations remained. The authors then demonstrated a second OR expressed primarily in antennae that responded to high concentrations of ( *S*)-1-octen-3-ol. The involvement of this antennal OR in the repellency of octenol at high concentrations is postulated. This is an intriguing paper that expands our knowledge octenol reception in the southern house mosquito. Since the antennal OR postulated for octenol reception is activated only at relatively high concentrations (10 ^-5^M and above), it would be interesting to discover its natural ligand.

I have read this submission. I believe that I have an appropriate level of expertise to confirm that it is of an acceptable scientific standard.
